# Divergence-degenerate spatial multiplexing towards future ultrahigh capacity, low error-rate optical communications

**DOI:** 10.1038/s41377-022-00834-4

**Published:** 2022-05-19

**Authors:** Zhensong Wan, Yijie Shen, Zhaoyang Wang, Zijian Shi, Qiang Liu, Xing Fu

**Affiliations:** 1 Key Laboratory of Photonic Control Technology (Tsinghua University), Ministry of Education, 100084 Beijing, China; 2 State Key Laboratory of Precision Measurement Technology and Instruments, Department of Precision Instrument, Tsinghua University, 100084 Beijing, China; 3 Optoelectronics Research center, University of Southampton, Southampton, SO17 1BJ UK

**Keywords:** Fibre optics and optical communications, Optical manipulation and tweezers

## Abstract

Spatial mode (de)multiplexing of orbital angular momentum (OAM) beams is a promising solution to address future bandwidth issues, but the rapidly increasing divergence with the mode order severely limits the practically addressable number of OAM modes. Here we present a set of multi-vortex geometric beams (MVGBs) as high-dimensional information carriers for free-space optical communication, by virtue of three independent degrees of freedom (DoFs) including central OAM, sub-beam OAM, and coherent-state phase. The novel modal basis set has high divergence degeneracy, and highly consistent propagation behaviors among all spatial modes, capable of increasing the addressable spatial channels by two orders of magnitude than OAM basis as predicted. We experimentally realize the tri-DoF MVGB mode (de)multiplexing and data transmission by the conjugated modulation method, demonstrating lower error rates caused by center offset and coherent background noise, compared with OAM basis. Our work provides a potentially useful basis for the next generation of large-scale dense data communication.

## Introduction

Multiplexing of independent optical degrees of freedom (DoFs) such as polarization and wavelength have long been implemented to increase the capacity of optical communication systems^1–4^. The exploration of spatial DoFs of optical fields has offered new possibilities that mode-division-multiplexing (MDM) scales the capacity by a factor equal to the number of spatial modes acting as independent information channel carriers^5–9^. Among all spatial modes, the use of orbital angular momentum (OAM) beams, which can accommodate theoretically infinite orthogonal modes, has generated widespread and significant interest in the last decade^10–18^. However, in practice, OAM modal basis set alone cannot reach the capacity limit of a communication channel^19^, since the beam diverges rapidly as the OAM order enlarges, which gives rise to increased power loss for a limited-size receiver aperture. To guarantee sufficient received optical power for data recovery, the number of OAM modes that can be practically supported is severely limited under 60^20,21^, mostly under 20^22–29^. One can relax the limit and increase the maximum number of addressable spatial channels, by enhancing the divergence degeneracy, i.e. having more orthogonal spatial modes propagating in identical manner. To this end, incorporating both the radial and azimuthal components of Laguerre–Gaussian (LG) beams^30–33^ makes one constructive step, but the improvement is far from satisfactory. To meet the growing demand for data capacity, it is highly desirable to use a large set of spatial modes with the variation in beam quality among all modes as small as possible.

In recent years, a class of exotic structured optical fields termed ray-wave geometric beams has attracted much attention, whereby crafted spatial modes appear to be both wave-like and ray-like^34–44^. In the wave picture, the beam is a coherent laser mode, imbued with a typical OAM feature. In the ray picture, the mode is coupled with a cluster of geometric rays, unveiling new controllable DoFs that notably increase the divergence degeneracy, such as sub-OAM (partial vortex along each ray) and coherent-state phase (the phase to tune the ray sequence).

In this work, we demonstrate that the modal basis of ray-wave geometric beams outperforms the OAM and LG modal basis, in terms of approaching the capacity limit of a free-space optical communication channel. Specifically, we create a three-dimensional set of orthogonal data-carrying beams, by employing three independent intrinsic DoFs of the multi-vortex geometric beam (MVGB), one type of ray-wave geometric beams, including the central OAM, sub-beam OAM and coherent-state phase. We show the MVGB set is extremely densely packed in beam quality space and has a highly consistent propagation behavior that it can possess a divergence degeneracy as high as 20, a 20X increase over OAM modal basis, and a divergence variation by merely 18% among 100 independent lowest order spatially multiplexed modes, in contrast to 900% for OAM counterpart and 429% for LG counterpart. As a result, thousands of independently spatial channels in MVGB basis can be supported in a free-space optical communication system, two orders of magnitude larger than that in OAM basis. To validate the performance of the high-dimensional information carriers, we analyze in detail the orthogonality of MVGB mode on different spatial indices, based on which we experimentally realize the tri-DoF mode (de)multiplexing and shift-keying encoding/decoding by the conjugated modulation using digital micro-mirror device (DMD). The results also indicate another distinct advantage of MVGB basis in demultiplexing with much lower bit error rate (ER) caused by the center offset and the coherent background noise, and thus having lower pixel ER in free-space data transmission, compared with OAM basis. We believe the divergence-degenerate MVGB modal set provides a useful basis for boosting the capacity of future optical communication systems.

## Results

### Tri-DoF MVGBs

The ray-wave geometric beam can be represented as the superposition of a family of eigenstates (Hermite-Laguerre-Gaussian (HLG) modes) with sub-Poissonian distribution:

1 $$\left| {{{\Psi }}_{n_0,m_0}^{(\alpha ,\beta ,\phi )}} \right\rangle _{p,q}^N = \frac{1}{{2^{N/2}}}\mathop {\sum}\limits_{K = 0}^N {\left( {\frac{N}{K}} \right)^{1/2}{\rm{e}}^{{\rm{i}}K\phi }{{{\mathrm{HLG}}}}_{n_0 + pK,m_0 + qK}^{(\alpha ,\beta )}}$$

where *N* + 1 is the number of eigenmodes in the frequency-degenerate family of ${{{\mathrm{HLG}}}}_{n_0 + pK,m_0 + qK}^{\left( {\alpha ,\beta } \right)}$, *p* and *q* are ratios of transverse frequency spaces in the *x*- and *y*-axis, respectively, *n*_0_ and *m*_0_ are the initial orders of transverse mode in the *x*- and *y*-axis respectively, and *ϕ* is the coherent-state phase. In particular, when *α* = *β* = ±*π/*2, the HLG eigenmode degenerates to LG mode^44^.

**Fig. 1:**
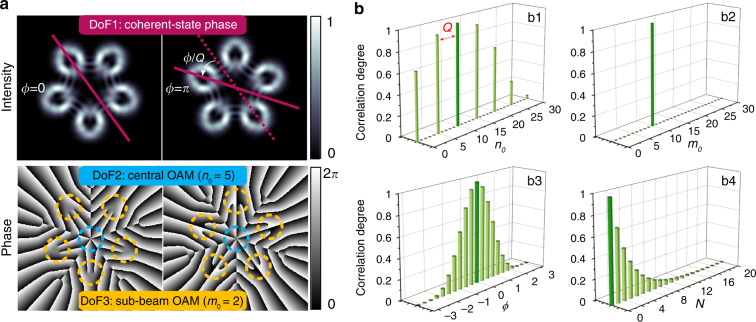
Illustration of tri-DoF MVGBs. **a** Diagram of three DoFs of MVGB $\left| {{{\Psi }}_{7,2}^{(\pi /2,\pi /2,\phi )}} \right\rangle _{5,0}^5$. **b** correlation degree analysis of MVGBs. **b1** correlation degree between $\left| {{{\Psi }}_{10,0}^0} \right\rangle ^5$ and$\left| {{{\Psi }}_{n_0,0}^0} \right\rangle ^5$ where *n*_0_ is changing from 0 to 30, showing that the order spacing of the non-orthogonal mode is *Q*, (**b2**) correlation degree between $\left| {{{\Psi }}_{5,10}^0} \right\rangle ^5$ and $\left| {{{\Psi }}_{5,m_0}^0} \right\rangle ^5$where *m*_0_ is changing from 0 to 25, showing that all modes are orthogonal to each other, (**b3**) correlation degree between $\left| {{{\Psi }}_{5,10}^0} \right\rangle ^5$ and $\left| {{{\Psi }}_{5,10}^{\pi /2}} \right\rangle ^N$ where *ϕ* is changing from −*π* to *π*, (**b4**) correlation degree between $\left| {{{\Psi }}_{5,10}^0} \right\rangle ^5$ and $| {{{\Psi }}_{5,10}^{\pi /2}} \rangle ^N$ where *N* is changing from 0 to 20. The dark bar in each subfigure indicates the reference index value for the correlation degree analysis

For the case of *p* = *Q* and *q* = 0, the ray-wave geometric beam is referred to as MVGB, having *Q* vortex sub-beams. In a MVGB, the coherent-state phase *ϕ* acting as one DoF is manifested in the orientation of petal-like intensity pattern, as shown in Fig. 1a that the rotation of orientation angle relative to the case of *ϕ* = 0 is *ϕ/Q*. The other two DoFs to be exploited are *n*_0_ and *m*_0_, the values of central OAM and sub-beam OAM, respectively, as demonstrated in the phase distribution in Fig. 1a. Hereinafter, we focus on the MVGB with *Q* = 5 as an example, the expression of which is thus abbreviated as $\left| {{{\Psi }}_{n_0,m_0}^\phi } \right\rangle ^N$ for the sake of brevity.

Before the demonstration of potential use of MVGBs in MDM communication application, it is crucial to first investigate the orthogonality of MVGBs in terms of three spatial indices, using the correlation degree as the metric. The correlation degree of 0 and 1 represent orthogonal and completely non-orthogonal conditions for two MVGBs, respectively. The correlation degree of two MVGBs with different parameters *n*_0_, *m*_0_ and *ϕ*, as represented by the inner product mathematically, is given by

2 $$\begin{array}{l}^N\left\langle {{{\Psi }}_{n_{0_s},m_{0_s}}^{\phi _s}} \right|\left. {{{\Psi }}_{n_{0_r},m_{0_r}}^{\phi _r}} \right\rangle ^N\\ = {\iint} {\frac{1}{{2^{N/2}}}} \mathop {\sum}\limits_{J = 0}^N {\left( {\begin{array}{*{20}{c}} N \\ J \end{array}} \right)^{1/2}} {{{\mathrm{e}}}}^{{{{\mathrm{ - i}}}}J\phi _s}\widetilde {{{{\mathrm{HLG}}}}}_{n_{0_s} + pJ,m_{0_s} + qJ}^{\left( {\pi /2,\pi /2} \right)}\\ \times \frac{1}{{2^{N/2}}}\mathop {\sum}\limits_{K = 0}^N {\left( {\begin{array}{*{20}{c}} N \\ K \end{array}} \right)^{1/2}} {{{\mathrm{e}}}}^{{{{\mathrm{i}}}}K\phi _r}{{{\mathrm{HLG}}}}_{n_{0_r} + pK,m_{0_r} + qK}^{\left( {\pi /2,\pi /2} \right)}{\rm{d}}x{\rm{d}}y\end{array}$$

where sign ’∼’ meansconjugate, and ${\iint} {\widetilde {{{{\mathrm{HLG}}}}}_{n_s,m_s}^{\left( {\pi /2,\pi /2} \right)} \times {{{\mathrm{HLG}}}}_{n_r,m_r}^{\left( {\pi /2,\pi /2} \right)}{\rm{d}}x{\rm{d}}y} = \delta _{(n_s,n_r),(m_s,m_r)}$(where $\delta _{(n_s,n_r),(m_s,m_r)} \,\ne\, 0$ only when *ns* = *nr* and *m*_*s*_ = *m*_*r*_).

According to Eq. (2), the theoretical results of the correlation degree analysis of MVGBs are shown in Fig. 1b. Basically, three spatial indices (*n*_0_, *m*_0_, and *ϕ*) are uncoupled and independent of each other. The orthogonalities of two MVGBs associated with each of three spatial indices are examined as follows. First, two MVGBs with different central OAM values (*n*_0_) are mutually orthogonal to each other when |*n*_0*s*_−*n*_0*r*_ | ≠ *ZQ* (*Z* is an integer and ${Z}\, \leqslant \, {N}$), as shown in Fig. 1b1 that the order spacing of the non-orthogonal mode is *Q*. Second, since *q* = 0 for MVGBs, it is natural that MVGBs with different sub-beam OAM values (*m*_0_) are orthogonal to each other, same as the case of general OAM beams, as illustrated in Fig. 1b2. Third, two MVGBs with the coherent-state phase (*ϕ*) being 0 and *π* respectively are orthogonal, which is analogous to the case of left- and right-hand circular polarization states, as depicted in Fig. 1b3. Furthermore, note that two MVGBs can be regarded as quasi-orthogonal when |*ϕ*_*s*_ − *ϕ*_*r*_| = *π/*2 (*N* > 5), since the correlation degree is less than 0.1, as described in Fig. 1b4. Therefore, *ϕ* can take up to four values (0*, π/*2*, π*, and 3*π/*2) when *N* > 5, for the realization of efficient mode (de)multiplexing. So far, we have obtained the MVGB modal basis set characterized by three spatial indices of *n*_0_, *m*_0_, and *ϕ*, enabling a combination of 4 × *K*_*n*_ × *K*_*m*_ readily available spatial modes as information carriers, where *K*_*n*_ and *K*_*m*_ are the numbers of central OAM and sub-beam OAM states selected from theoretically unbounded states respectively. The detailed orthogonality analysis of general ray-wave geometric beam can be found in Supplementary Note 1.

### High divergence degeneracy

The beam propagation dynamics in free space is vital for free-space optical communication^45^ and governed by the beam quality factor *M*^2^ entirely. The dynamic transmission characteristics of the beam include the beam size and divergence angle, where the divergence angle of the beam determines the transverse spatial frequency of the beam. For a LG_*pl*_ mode and a MVGB mode as a superposition of multiple higher-order eigenmodes (${{{\mathrm{HLG}}}}_{n,m}^{(\alpha ,\beta )}$), the beam quality factors are respectively expressed as^46^

3 $$M_{{{{\mathrm{LG}}}}}^2 = 2p + \left| l \right| + 1$$

4 $$M_{{{{\mathrm{MVGB}}}}}^2{{{\mathrm{ = }}}}\mathop {\sum}\limits_{m = 0}^\infty {\mathop {\sum}\limits_{n = 0}^\infty {\left( {n + m + 1} \right)\left| {c_{nm}} \right|^2} }$$

where $$c_{nm} = \frac{1}{{2^{N/2}}}\left( {\begin{array}{*{20}{c}} N \\ K \end{array}} \right)^{1/2}$$ are normalized amplitudes for the eigenmodes ${{{\mathrm{HLG}}}}_{n_0 + QK,m_0}^{\left( {\alpha ,\beta } \right)}$ with *n* = *n*_0_ + *QK* and *m* = *m*_0_ in Eq. (1), and the total power $$\mathop {\sum}\nolimits_{m = 0}^\infty {\mathop {\sum}\nolimits_{n = 0}^\infty {\left| {c_{nm}} \right|^2} } = \frac{1}{{2^{N/2}}}\mathop {\sum}\nolimits_{K = 0}^N {\left( {\begin{array}{*{20}{c}} N \\ K \end{array}} \right)^{1/2}} = 1$$

It can be seen from Eq. (4) that the beam quality factor of the MVGB depends entirely on the family of eigenmodes it contains and the corresponding normalized weighting factor. Notably, the coherent-state phase parameter does not affect the superposition components and weighting factors, thus the additional DoF of *ϕ* can scale the beam quality degeneracy by a factor of 4 (equal to the number of employed values of *ϕ*), compared with two-dimensional LG modal basis. For instance, as Fig. S3 shows, among the 100 lowest orders of MVGB modes, by combinations of *m*_0_ = {0,1,2,3,4}, *n*_0_ = {0,1,2,3,4}, and *ϕ* = {0*,π/*2*,π*,3*π/*2}, the maximum beam quality degeneracy reaches as high as 20, that up to 20 modes share the same beam quality factor of *M*^2^ = 17.5. This leads to only a total of 9 beam quality factors from all the 100 modes: *M*^2^ = {13.5,14.5,15.5,16.5,17.5,18.5,19.5,20.5,21.5}. In contrast, the 100 lowest orders of LG_*pl*_ modes, by combinations of *p* and *l* both taking 10 integer values from 0 to 9, have 28 integer values of *M*^2^ from 1 to 28.

Similarly, the divergence and beam waist diameter of MVGBs have high degeneracies. The beam size can be calculated by the second moment of intensity^47^, thus we have the beam waist diameter *D*_0*,m*_ and divergence *θ*_*m*_ of m-order LG modes and MVGBs expressed as:

5 $$\begin{array}{l}D_{0,m} = 2\sqrt {\frac{{2{\int}_0^{2\pi } {{\int}_0^\infty {r^2I_0(r,\phi )r{\rm{d}}r{\rm{d}}\phi } } }}{{{\int}_0^{2\pi } {{\int}_0^\infty {I_0(r,\phi )r{\rm{d}}r{\rm{d}}\phi } } }}} \\ {{{\mathrm{ }}}}\theta _m = \frac{{M^2\lambda }}{{\pi D_{0,m}}}\end{array}$$

where *I*_0_(*r, ϕ*) is the intensity distribution of beam waist cross-section.

**Fig. 2:**
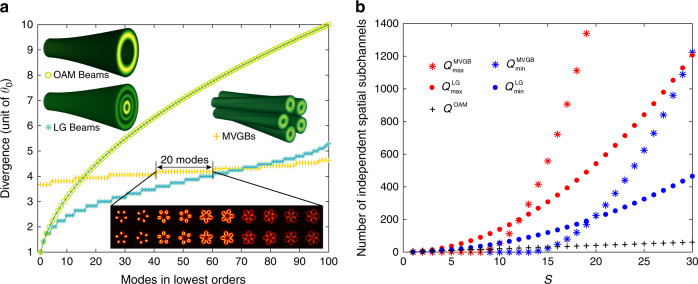
High divergence degeneracy of MVGBs. **a** Comparison on divergences of MVGBs, OAM modes and LG modes, in respective 100 lowest orders. Insets illustrate three-dimensional diagrams of three types of modes, as well as the transverse intensity profiles of 20 MVGB modes in degenerated divergence. **b** Comparison on predicted numbers of independently addressable spatial subchannels in a spatial multiplexing system using MVGBs, OAM modes and LG modes, where the dimensionless parameter *S* is equal to *π*/4 times the space–bandwidth product (SBP)

Figure 2a compares the divergences of OAM modes, LG modes and MVGBs in respective 100 lowest orders, all normalized to the divergence angle of fundamental Gaussian beam (*θ*_0_). Note that the maximum divergence degeneracy is 20, that 20 modes share the same divergence of *θ*_*m*_ = 4.18 *θ*_0_, and the divergence varies by merely 18% among 100 independent lowest order spatially multiplexed modes of MVGBs, in contrast to 900% for OAM counterpart and 429% for LG counterpart. This brilliant feature of MVGB basis results in a highly consistent propagation behavior of data channels, which is beneficial for the beam tracking, and alignment control of receiver optics and adaptive optics^6^. The corresponding variations in beam waist diameter for LG modes and MVGBs are compared in Fig. S4.

By the virtue of high divergence degeneracy, we claim that the MVGB basis can achieve capacity beyond OAM and LG counterparts. To confirm this, we count the number of MVGB modes that fit into a line-of-sight free-space communication system with a space–bandwidth product (SBP) of 2*R*_0_ × 2NA*/λ*, where *R*_0_ and NA are the aperture radius and numerical aperture of both circular apertures of transmitter and receiver, and *λ* is the wavelength. Following the procedure of ref. ^19^, we define a dimensionless parameter *S*, which is *π/*4 times the SBP, and then estimate the lower and upper bound on the number of independently addressable spatial subchannels *Q*, counting all MVGB modes that satisfy $M_{{{{\mathrm{MVGB}}}}}^2 \, \leqslant \, S$, as given by

6 $$\begin{array}{l}Q_{\min }^{MVGB} = 8 \times \frac{{\left \lfloor {S - 12.5} \right\rfloor \times \left( {\left\lfloor {S - 12.5} \right\rfloor + 1} \right)}}{2}\\\qquad\quad {{{\mathrm{ }}}} \,\ge\! 4\left( {S - 13} \right)\left( {S - 12} \right)\end{array}$$

where ⌊⌋ represents the floor integration.

7 $$Q_{\max }^{MVGB} \ge\! 4\left( {\frac{{16S}}{{\pi ^2}} - 13} \right)\left( {\frac{{16S}}{{\pi ^2}} - 12} \right)$$

The estimated subchannel numbers of MVGB multiplexing in an SBP-limited free-space optical communication system based on Eq. 6 and Eq. 7 are compared with those of OAM multiplexing and LG mode multiplexing in Fig. 2b. It is noteworthy that although the lowest beam quality of MVGB is higher than OAM beams and LG modes, corresponding to a larger intercept in the x-axis, the lower and upper bound curves of MVGB have far steeper slopes versus *S* (equivalently the SBP) than OAM and LG modal basis, thereby accomodating far more data channels. For example, at *S* = 30 (SBP = 38), *Q*^OAM^ is about 60, *Q*^LG^ lies between 460 and 1200, and *Q*^MVGB^ lies between 1200 and 5000, which is about two orders of magnitude larger than OAM basis. The superiority of MVGB modal basis would become even more distinct, in terms of addressable independent spatial subchannels, when the communication system has higher SBP and larger scales, as indicated by the trend in Fig. 2b. The free-space propagating performance of MVGBs in the presence of atmospheric turbulence is investigated in Supplementary Note 2.

### Low ER in MVGB demultiplexing

**Fig. 3:**
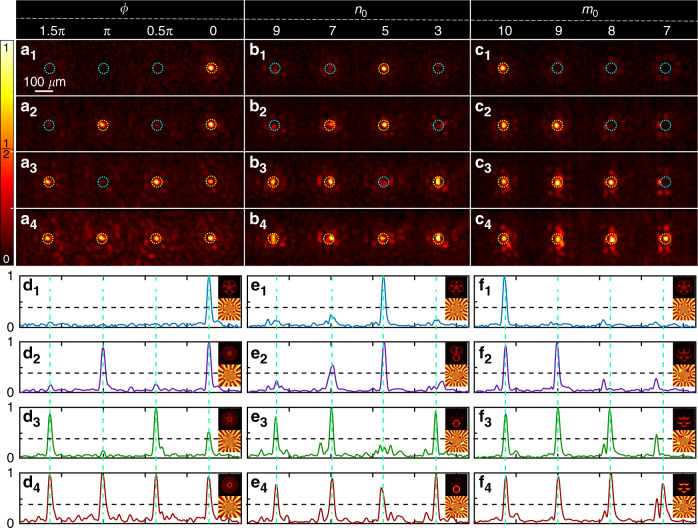
Tri-DoF demultiplexing results of MVGBs. Three demultiplexed conjugated holographic masks for DoFs of *ϕ, n*_0_*, m*_0_ are obtained from non-collinearly superposed conjugated optical field of MVGBs: ${\sum} {_{\phi = 0,\pi /2,\pi ,3\pi /2}\left| {\widetilde {{\Psi }}_{5,10}^\phi } \right\rangle ^5\exp \left( {{{{\mathrm{i}}}}2\pi u_\phi x} \right)}$, ${\sum} {_{n_0 = 3,5,7,9}\left| {\widetilde {{\Psi }}_{n_0,10}^0} \right\rangle ^5\exp } \left( {{{{\mathrm{i}}}}2\pi u_{n_0}x} \right)$ and ${\sum} {_{m_0 = 7,8,9,10}\left| {\widetilde {{\Psi }}_{5,m_0}^0} \right\rangle ^5\exp \left( {{{{\mathrm{i}}}}2\pi u_{m_0}x} \right)}$. (**a**_**1**_–**a**_**4**_), (**b**_**1**_–**b**_**4**_) and (**c**_**1**_–**c**_**4**_) are experimental intensity profiles of demultiplexed beam components for incident light with one to four collinearly superposed MVGB on the DoFs of *ϕ*, *n*_0_ and *m*_0_, respectively. (**d**_**1–4**_) to (**f**_**1–4**_) are corresponding one dimensional cross-section views of (**a**_**1–4**_) to (**c**_**1–4**_), where the insets are the intensity and phase distribution of the corresponding input fields. Black dash line: discrimination threshold (DT)

**Fig. 4:**
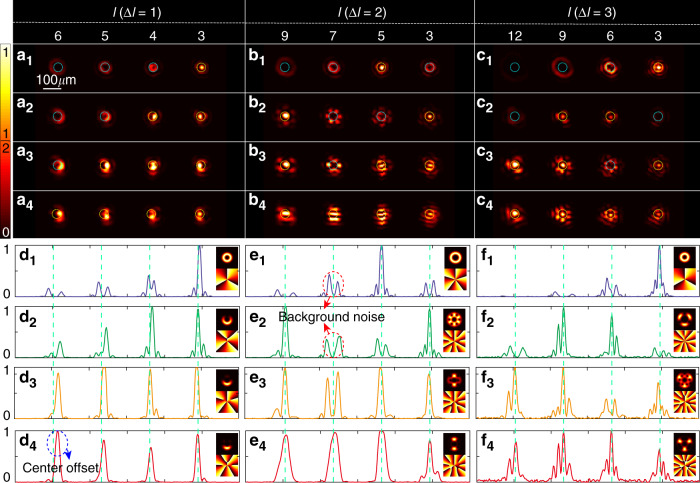
Demultiplexing results of OAM beams. Three demultiplexed conjugated holographic masks for OAM mode spacing of 1, 2 and 3 are obtained from non-collinearly superposed conjugated optical field of MVGBs: ${\sum} {_{l = 3,4,5,6}\widetilde {{{{\mathrm{LG}}}}}_{p = 0}^l\exp \left( {{{{\mathrm{i}}}}2\pi u_lx} \right)}$, ${\sum} {_{l = 3,5,7,9}\widetilde {{{{\mathrm{LG}}}}}_{p = 0}^l\exp \left( {{{{\mathrm{i}}}}2\pi u_lx} \right)}$ and ${\sum} {_{l = 3,6,9,12}\widetilde {{{{\mathrm{LG}}}}}_{p = 0}^l\exp \left( {{{{\mathrm{i}}}}2\pi u_lx} \right)}$. (**a**_**1**_–**a**_**4**_), (**b**_**1**_–**b**_**4**_) and (**c**_**1**_–**c**_**4**_) are experimental intensity profiles of demultiplexed beam components for incident light with one to four collinearly superposed OAM beams with ∆*l* = 1, ∆*l* = 2 and ∆*l* = 3, respectively. (**d**_**1–4**_) to (**f**_**1–4**_) are corresponding one dimensional cross-section view of (**a**_**1–4**_) to (**c**_**1–4**_), where the insets are the intensity and phase distribution of the corresponding input fields

Despite the widely applied sorting approaches developed for OAM beams and LG beams, such as Dammann vortex grating^23,48,49^, Gouy phase radial mode sorter^50^, log-polar based azimuthal mode sorters^51,52^, interference and diffraction method^53–55^, and deep learning^56^, identification and sorting of ray-wave geometric beams such as MVGBs is still at its infancy, due to the intrinsic complex structure and rich controlling parameters. Inspired by the recent work of digital cavity-free tailoring^44^, here we demonstrate the sorting and demultiplexing of superposed MVGBs in the following experiments, using the demultiplexed conjugated holographic masks that are designed by diffracting each beam component into different location^57,58^, as detailed in “Materials and methods”. Figure 3 shows the experimental results of MVGB demultiplexing associated with each of three spatial indices. For the DoF of *ϕ*, subfigures a_1_ to a_4_ demonstrate the intensity profiles of demultiplexed beam components separated along the *x* direction. The corresponding input of collinearly superposed MVGBs containing one to four beam components are ${\sum} {_{\phi = 0}\left| {{{\Psi }}_{5,10}^\phi } \right\rangle ^5}$, ${\sum} {_{\phi = 0,\pi }\left| {{{\Psi }}_{5,10}^\phi } \right\rangle ^5}$, ${\sum} {_{\phi = 0,\pi /2,3\pi /2}\left| {{{\Psi }}_{5,10}^\phi } \right\rangle ^5}$and ${\sum} {_{\phi = 0,\pi /2,\pi ,3\pi /2}\left| {{{\Psi }}_{5,10}^\phi } \right\rangle ^5}$, respectively. Corresponding demultiplexed conjugated holographic masks are designed as $T(x,y) = \frac{1}{2} + \frac{1}{2}{{{\mathrm{sign}}}}\left[ {\cos \left( \Phi \right) + \cos \left( {{{{\mathrm{arcsin}}}}A} \right)} \right]$, where *A* and Φ are respectively the amplitude and phase of non-collinearly superposed conjugated optical field of MVGBs ${\sum} {_{\phi = 0,\pi /2,\pi ,3\pi /2}\left| {\widetilde {{\Psi }}_{5,10}^\phi } \right\rangle ^5\exp \left( {{{{\mathrm{i}}}}2\pi u_\phi x} \right)}$ (see details in “Materials and methods”). The four dotted circles in each subfigure of a_1–4_ indicate the target diffracting locations of all four beam components by the holographic mask design, among which the yellow ones are signal locations, corresponding to those beam components that are practically multiplexed in the input beams, and the blue ones are non-signal locations. The same method is applied to OAM demultiplexing for the comparison in the next subsection, as shown in Fig. 4. Moreover, an 8-bit and 16-bit hybrid shift-keying encoding/decoding scheme with tri-DoF MVGBs are demonstrated with zero bit ER, as detailed in Supplementary Note 3. Another advantage of MVGB multiplexing is manifested in the low bit ER in the demultiplexing process of conjugated modulation.

Coherent light sources are widely exploited in MDM communication, since the mode coding can be done by a modulation device and does not require the spatial coupling. However, coherence may bring difficulty to signal decoding and thus increase the bit ER, which is defined as the proportion of bit values that are incorrectly identified according to the demultiplexing results. On the one hand, the intensity peak of a coherently superposed beam may deviate from the co-propagating optical axis, leading to the center offset, lateral displacement of intensity peak relative to the target location, of decoding spot and thus possibly introducing a bit error that the bit value of 1 is incorrectly identified as 0, e.g. the marked peak in Fig. 4(d_4_) for the case of OAM beams. The bit ER caused by center offset depends on the radius of the discrimination region (RD) that the bit error induced by center offset is valid only when the offset is larger than the RD. On the other hand, the background noise embedded in different beam components may be coherently superposed near the target diffracting position, resulting in a noise peak beyond the discrimination threshold (DT) that may introduce a bit error that bit value of 0 is incorrectly identified as 1, e.g. the marked peaks in subfigures e_1_ and e_2_ of Fig. 4. The bit ER caused by background noise depends on DT that a bit error is valid when the intensity peak of background noise is higher than DT in the discrimination region.

We emphasize that MVGBs have remarkably stronger capability, in contrast to OAM beams, in suppressing the bit ER caused by center offset and non-negligible background noise in the demultiplexing process of coherent light, thereby efficiently improving the signal-to-noise ratio and reducing the inter-modal crosstalk. First, due to the inherently more complicated intensity and phase distribution of MVGB than OAM beam, the intensity profile of superposed MVGBs tends to maintain the centrosymmetric feature especially on the DoF of *ϕ*, which is vital in mitigating the center offset. Second, for the input beam components that do not match with the demultiplexed mask, the complex phase structure of MVGB enables the responses at the non-signal locations as weak dispersing speckles (see Fig. 3), rather than concentrated spot patterns which are common in the case of OAM beams (see Fig. 4).

**Fig. 5:**
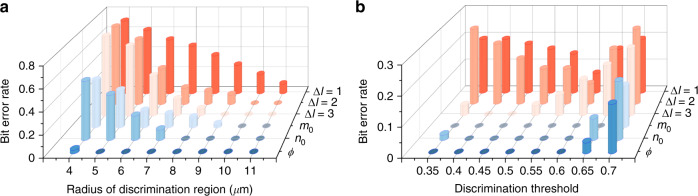
Low ER in MVGB demultiplexing. The bit ER caused by center offset (**a**) and background noise (**b**) in the demultiplexing process: comparison among the OAM beams with different mode spacings and tri-DoF MVGBs. of OAM beams (see Fig. 4)

To further verify the superiority, we compare the demultiplexing performance of MVGBs and OAM beams in terms of the measured bit ERs caused by center offset and background noise, as shown in Fig. 5a, b, respectively. The average offsets of the MVGBs on three DoFs (*ϕ, n*_0_*, m*_0_) are 2.69 *µ*m, 4.47 *µ*m, and 3.95 *µ*m, respectively, while those for OAM beams (mode spacing of ∆*l* = 1, 2, and 3) are 6.62 *µ*m, 5.31 *µ*m, and 5.25 *µ*m, respectively. As a result, the bit ER averaging among all values of RD (from 4 *µ*m to 11 *µ*m) are 0.16, 0.37, and 0.31 for MVGBs (*ϕ, n*_0_*, m*_0_), and are 0.59, 0.48, and 0.46 for OAM beams (∆*l* = 1, 2, and 3), respectively, as shown in Fig. 5a. It is of particular interest that MVGBs on the DoFs of *ϕ* and *n*_0_ have zero bit ER with RD above 5 *µ*m and 8 *µ*m, respectively. Figure 5b shows the bit ER results merely induced by the background noise, in which a large RD as 30 *µ*m is used to prevent the influence of center offset on the bit ER. It is impressive that MVGB has zero bit ER for demultiplexing on all three DoFs at ${0.4}\,\leqslant\,{\mathrm{DT}}\,\leqslant\,{0.6}$, in which the lower bound of zero bit ER for the case of *ϕ* can reach as low as 0.2 (not shown in the plot). In contrast, demultiplexing of OAM beams yields a high bit ER by background noise, which increase from 0.071 with DT = 0.6 to 0.196 with DT = 0.4, for the cases of OAM mode spacing of 1. When the OAM mode spacing increases to 3, the bit ER reduces to zero but at a narrow DT range $({0.4}\,\leqslant\,{\mathrm{ST}}\,\leqslant\,{0.45})$. Note that the bit ER increases rapidly for all the cases with DT higher than 0.6, which is not caused by an extremely high noise level beyond DT, but by the uneven intensity responses among different beam components of signal, causing that certain beam component has a smaller signal intensity than DT and the bit value at corresponding channel is incorrectly identified as 0. All these experimental results show that the tri-DoF MVGBs outperform the general OAM beams in terms of low-bit ER, indicating that tri-DoF MVGBs are advantageous as potential high-dimensional information carriers.

**Fig. 6:**
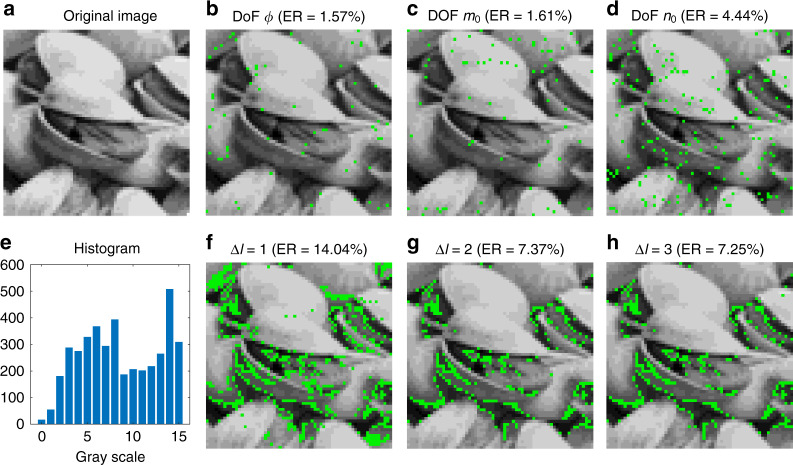
Transmitted image data. **a** 4-bit original image. **b**–**d** Retrieved image by the carriers of tri-DoF MVGBs. **e** 16-level grayscale histogram. **f**–**h** Retrieved image by the carriers of OAM beams with different mode spacings. The green pixels indicate the incorrect data received

Lastly, we demonstrate the data transmission by shift-keying method using the tri-DoF MVGBs. The data packet used is a 4-bit 16-level grayscale image composed of 64 × 64 pixels with an equalized gray-level histogram, as shown in Fig. 6a, e. We use four modes by shift-keying coding that the 4-bit grayscale information of each pixel is fully encoded into one pulse. For instance, the grayscale ^′^0101^′^ is encoded in a collinearly superposed MVGBs ${\sum} {_{\phi = 0,\pi }| {{{\Psi }}_{5,10}^\phi } \rangle ^5}$ on the DoF of *ϕ* (see details in “Materials and methods”). We decode the received information with RD = 11 *µ*m and DT = 0.5. The reconstructed images using MVGB carriers in three DoFs (*ϕ, n*_0_*, m*_0_) as shown in Fig. 6b–d, are compared with that using OAM beam carriers with different mode spacings (∆*l* = 1, 2, and 3) as shown in Fig. 6f–h, in which the pixels that receive incorrect gray-level information are marked in green. It can be seen in Fig. 6 that the tri-DoF MVGB carrier reliably transmits the image, outperforming the general OAM beams in terms of pixel ER. In addition, the pixel ER measured for the MVGB case matches the bit ERs results for DT = 0.5 in Fig. 5b.

## Discussion

The emergence of structured light offers a possible solution to meet future demands for communication capacity, by utilizing its spatial DoFs from high-dimensional orthogonality. In this work, we introduce a novel modal basis of MVGBs with three DoFs including the central OAM, sub-beam OAM and coherent-state phase. At the heart of our work is the exploitation of modes in ray-wave duality state, which allows us access to higher divergence degeneracy and more consistent propagation behavior among all modes, dramatically increasing the addressable number of independent spatial channels. We validate the potentials of spatially multiplexed MVGB as high-dimensional information carriers, by proof-of-concept experiments of the tri-DoF mode (de)multiplexing and shift-keying encoding/decoding. Notably, we make the challenging demultiplexing task of tri-DoF modes possible, by proposing a novel approach based on conjugated modulation that can fully resolve the ray-wave duality state, removing the long-standing obstacle in the identification and sorting of ray-wave geometric beam that has prohibited its progress. The decoding results show that MVGB modal basis has significant strengths in suppressing bit ERs induced by center offset and coherent background noise, leading to successful data-packet transmission with much lower pixel ER than OAM beams.

The MVGB multiplexing is compatible with and can combine with other techniques, such as wavelength and polarization division multiplexing, and may also work within fibers, to further increase capacity and facilitate the implementation of next-generation high-capacity free-space communication network. Our technique could be extended to other types of ray-wave geometric beams, to explore even more spatial DoFs and higher divergence degeneracy. The concept of tri-DoF modal basis can also be applied to encoding and decoding in the quantum data channels. In the future, we will exploit the nonseparability among multiple DoFs to further explore its value in realizing high-dimensional multipartite entanglement.

## Materials and methods

### Experimental setup

**Fig. 7:**
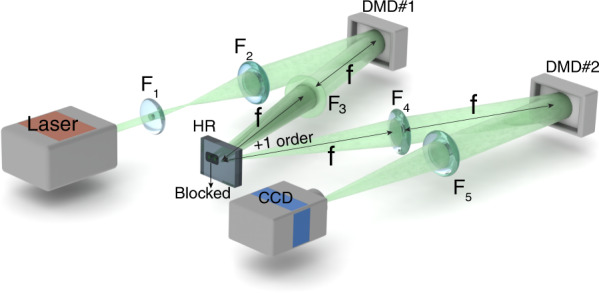
Schematic of experimental setup. *F*_1_−*F*_5_: lens, HR: High reflective mirror; CCD: charge coupled device (Microview RS-A1500-GM60 with a resolution of 1280 × 1024 pixels and a pixel size of 5.3 *µ*m), DMD: digital micro-mirror device (F6500 Type A 1080P VIS KIT, resolution: 1920 × 1080 pixels)

The experimental setup is shown in Fig. 7. The beam from a solid laser source (CNI laser, MGL-III-532nm) is expanded to a near-plane wave by passing through a telescope (*F*_1_, focal length of 25 mm; *F*_2_, focal length of 300 mm) with the magnification of 1:12, and illuminates DMD #1 loaded with a hologram of the target light field. Then the first order of the beam is selected and reflected by a high reflective (HR) mirror, image relayed by a 4*f* system with F_3_ and F_4_, both with the focal length of 150 mm, and transmitted to DMD#2 loaded with holograms of corresponding conjugated optical field. The modulated beam is focused into a spot by a convex lens (*F*_5_, focal length of 150 mm), the intensity profile of which is captured by a CCD camera located at the focal plane of *F*_5_.

The discrimination and measurement of the correlation degree of ray-wave geometric beams can be realized by mode projective measurement. The hologram for producing the input beams is loaded into DMD #1, then we load a series of the corresponding conjugated holographic mask into DMD #2 sequentially and capture the corresponding focal spots using CCD camera, which performs a modal decomposition on every DoF. The measured results of correlation degree are shown in Fig. S2. Meanwhile, in the demultiplexing experiments for MVGBs and OAM beams, DMD #1 is loaded with the hologram of the collinearly superposed light field for generating the multiplexed beam, and DMD #2 is loaded with the demultiplexed hologram for separating and identifying beam components in the multiplexed beam.

### Data transmission by shift-keying coding

Experiments of shift-keying data transmission rely on the demultiplexing results of MVGBs and OAM beams in Fig. 3 and Fig. 4. Taking the DoF of *ϕ* in Fig. 3 as an example, the states of 1.5*π*, *π*, 0.5*π*, and 0 from left to right correspond to the 4th, 3rd, 2nd, and 1st bit of a 4-bit binary signal, respectively. In mode shift-keying coding, *N* bits binary sequence corresponds to the collinear superposition coefficients of the beam components in the multiplexed MVGBs, that is, the corresponding MVGB component is presented when the signal bit value is 1, and absent when it is 0. Therefore, the results in Fig. 3(a_2_) correspond to ^′^0101^′^ in the binary system and ^′^5^′^ in the decimal system. The rest of the mode shift-keying process follows the same principle.

In the experiment, DMD #1 and #2 shown in Fig. 7 serve as the encoder and decoder respectively. First, a sequence of time-varying multiplexed MVGBs or OAM beams is obtained by the holograms loaded on DMD #1 according to the encoded signal. Then the encoded multiplexed beams propagate in the free space and illuminate DMD #2 loaded with a constant demultiplexed conjugated holographic mask, getting separated into 4 diffraction positions, with the focal spots recorded by CCD. Finally, we obtain the results in Fig. 6 by recovering the signal.

### Demultiplexing design of MVGBs

**Fig. 8:**
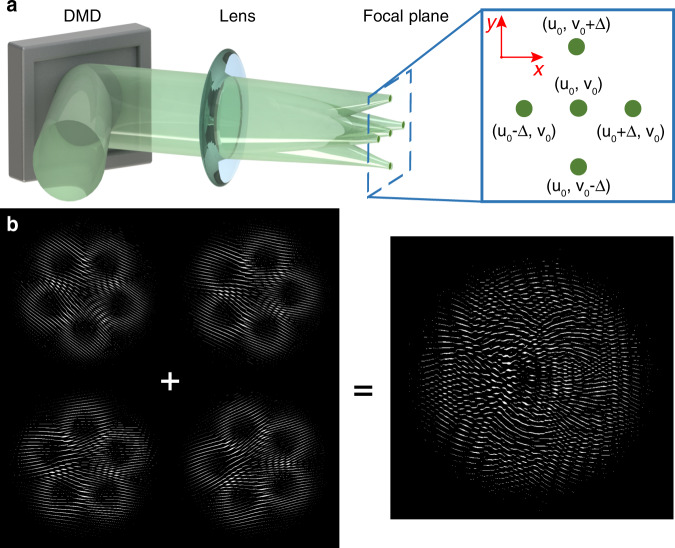
Demultiplexing design of MVGBs. **a** Diagram of beam spatial demultiplexing. The identified sub-beam can be spatially separated by adding a different linear grating to the corresponding conjugate optical field. **b** conjugated holographic masks of four identified beam components with different linear grating and demultiplexed conjugated holographic mask

The DMD transmission function of the hologram of conjugated optical field modulation is given as:

8 $$T_s\left( {x,y} \right) = M[A, - \Phi + 2\pi \left( {u_0x + v_0y} \right)]$$

where$M(\alpha ,\beta ) = \frac{1}{2} + \frac{1}{2}{{{\mathrm{sign}}}}\left[ {\cos \left( \beta \right) + \cos \left( {{{{\mathrm{arcsin}}}}\alpha } \right)} \right]$ (see details in Supplementary Note 2). The target diffracting position of a beam component is determined by the linear grating period (*u*_0_, *v*_0_). The demultiplexed conjugated holographic mask is calculated by a non-collinearly superposed conjugated optical field with different periods of linear grating, which means the diffraction direction of each beam component is separated, as shown in Fig. 8a. The non-collinearly superposed conjugated optical field of different linear grating to the corresponding conjugate optical field of MVGBs is:

9 $$CSU = \mathop {\sum}\limits_n {\widetilde {SU}_s^n} = \mathop {\sum}\limits_n {\widetilde {SU}^n\exp \left[ {{{{\mathrm{i}}}}2\pi \left( {u_nx + v_ny} \right)} \right]}$$

where $\widetilde {SU}^n$ are a set of orthogonal MVGB components. According to Eq. 8, different linear grating is added to each conjugate optical field, as described in Fig. 8b. The demultiplexed conjugated holographic mask can be obtained as:

10 $$T_s^D\left( {x,y} \right) = M\left( {A^D,\Phi ^D} \right)$$

where *A*^*D*^ and Φ^*D*^ are normalized amplitude and phase of *CSU*, respectively. The *u*_*n*_ and *v*_*n*_ are the reciprocal of the period of the linear grating in the *x* and *y* direction of the *n*th multiplexed mode, respectively.

## Supplementary information

Supplementary Information for Divergence-degenerate spatial multiplexing towards future ultrahigh capacity, low error-rate optical communications

## References

[1] Kaminow IP (1996). A wideband all-optical WDM network. IEEE J. Sel. Areas Commun..

[2] Hung, W. et al. An optical network unit for WDM access networks with downstream DPSK and upstream re-modulated OOK data using injection-locked FP laser. In: *OFC 2003 Optical Fiber Communications Conference,**2003*. 281–282 (IEEE, 2003).

[3] Chow CW, Wong CS, Tsang HK (2004). Optical packet labeling based on simultaneous polarization shift keying and amplitude shift keying. Opt. Lett..

[4] Mukherjee, B. *Optical WDM Networks* (Springer, 2006).

[5] Willner AE (2021). Orbital angular momentum of light for communications. Appl. Phys. Rev..

[6] Trichili A (2019). Communicating using spatial mode multiplexing: potentials, challenges, and perspectives. IEEE Commun. Surv. Tutor..

[7] Bozinovic N (2013). Terabit-scale orbital angular momentum mode division multiplexing in fibers. Science.

[8] Shen YJ (2019). Optical vortices 30 years on: OAM manipulation from topological charge to multiple singularities. Light.: Sci. Appl..

[9] Wang J (2016). Advances in communications using optical vortices. Photonics Res..

[10] Allen L (1992). Orbital angular momentum of light and the transformation of Laguerre-Gaussian laser modes. Phys. Rev. A.

[11] Fickler R (2012). Quantum entanglement of high angular momenta. Science.

[12] Fang XY (2021). High-dimensional orbital angular momentum multiplexing nonlinear holography. Adv. Photonics.

[13] Chen J (2021). Engineering photonic angular momentum with structured light: a review. Adv. Photonics.

[14] Fang XY, Ren HR, Gu M (2020). Orbital angular momentum holography for high-security encryption. Nat. Photonics.

[15] Willner AE (2021). Perspectives on advances in high-capacity, free-space communications using multiplexing of orbital-angular-momentum beams. APL Photonics.

[16] Mair A (2001). Entanglement of the orbital angular momentum states of photons. Nature.

[17] Erhard M (2018). Twisted photons: new quantum perspectives in high dimensions. Light.: Sci. Appl..

[18] Erhard M, Krenn M, Zeilinger A (2020). Advances in high-dimensional quantum entanglement. Nat. Rev. Phys..

[19] Zhao NB (2015). Capacity limits of spatially multiplexed free-space communication. Nat. Photonics.

[20] Wang, J. et al. N-dimentional multiplexing link with 1.036-Pbit/s transmission capacity and 112.6-bit/s/Hz spectral efficiency using OFDM-8QAM signals over 368 WDM pol-muxed 26 OAM modes. In: *2014 The European Conference on Optical Communication (ECOC)*, 1–3 (IEEE, 2014).

[21] Wang, J. et al. Ultra-high 435-bit/s/Hz spectral efficiency using N-dimentional multiplexing and modulation link with pol-muxed 52 orbital angular momentum (OAM) modes carrying Nyquist 32-QAM signals. In: *2015 European Conference on Optical Communication (ECOC)*, 1–3 (IEEE, 2015).

[22] Willner, A. E. et al. in *Electromagnetic Vortices* (eds. Jiang, Z. H. & Werner, D. H.) Ch. 12, 357–400 (John Wiley Sons, Ltd, 2021).

[23] Lei T (2015). Massive individual orbital angular momentum channels for multiplexing enabled by Dammann gratings. Light.: Sci. Appl..

[24] Wang J (2012). Terabit free-space data transmission employing orbital angular momentum multiplexing. Nat. Photonics.

[25] Krenn M (2014). Communication with spatially modulated light through turbulent air across Vienna. N. J. Phys..

[26] Wang ZX, Zhang N, Yuan XC (2011). High-volume optical vortex multiplexing and de-multiplexing for free-space optical communication. Opt. Express.

[27] Krenn M (2016). Twisted light transmission over 143km. Proc. Natl Acad. Sci. USA.

[28] Ren YX (2016). Experimental characterization of a 400 Gbit/s orbital angular momentum multiplexed free-space optical link over 120 m. Opt. Lett..

[29] Huang H (2014). 100 Tbit/s free-space data link enabled by three-dimensional multiplexing of orbital angular momentum, polarization, and wavelength. Opt. Lett..

[30] Li L (2017). Power loss mitigation of orbital-angular-momentum-multiplexed free-space optical links using nonzero radial index Laguerre–Gaussian beams. J. Optical Soc. Am. B.

[31] Guo ZY (2018). The orbital angular momentum encoding system with radial indices of Laguerre–Gaussian beam. IEEE Photonics J..

[32] Pang K (2018). 400-Gbit/s QPSK free-space optical communication link based on four-fold multiplexing of Hermite–Gaussian or Laguerre–Gaussian modes by varying both modal indices. Opt. Lett..

[33] Xie GD (2016). Experimental demonstration of a 200-Gbit/s free-space optical link by multiplexing Laguerre–Gaussian beams with different radial indices. Opt. Lett..

[34] Bužek V, Quang T (1989). Generalized coherent state for bosonic realization of SU(2)Lie algebra. J. Optical Soc. Am. B.

[35] Wodkiewicz K, Eberly JH (1985). Coherent states, squeezed fluctuations, and the *SU*(2) am *SU*(1, 1) groups in quantum-optics applications. J. Optical Soc. Am. B.

[36] Shen YJ (2018). Periodic-trajectory-controlled, coherent-state-phase-switched, and wavelength-tunable SU(2) geometric modes in a frequency-degenerate resonator. Appl. Opt..

[37] Shen YJ (2018). Polygonal vortex beams. IEEE Photonics J..

[38] Shen YJ, Fu X, Gong ML (2018). Truncated triangular diffraction lattices and orbital-angular-momentum detection of vortex SU(2) geometric modes. Opt. Express.

[39] Shen YJ (2021). Rays, waves, SU(2) symmetry and geometry: toolkits for structured light. J. Opt..

[40] Shen YJ (2020). SU(2) poincaré sphere: a generalized representation for multidimensional structured light. Phys. Rev. A.

[41] Chen YF (2006). Devil’s staircase in three-dimensional coherent waves localized on lissajous parametric surfaces. Phys. Rev. Lett..

[42] Lu TH (2008). Three-dimensional coherent optical waves localized on trochoidal parametric surfaces. Phys. Rev. Lett..

[43] Chen YF (2010). Spatial transformation of coherent optical waves with orbital morphologies. Phys. Rev. A.

[44] Wan ZS (2020). Digitally tailoring arbitrary structured light of generalized ray-wave duality. Opt. Express.

[45] Forbes, A. *Laser Beam Propagation: Generation and Propagation of Customized Light* (CRC Press, 2014).

[46] Siegman, A. E. New developments in laser resonators. In: *Proc. 1224 SPIE, Optical Resonators* 2-14 (SPIE, 1990).

[47] Willner AE (2016). Design challenges and guidelines for free-space optical communication links using orbital-angular-momentum multiplexing of multiple beams. J. Opt..

[48] Zhang N (2010). Analysis of multilevel spiral phase plates using a Dammann vortex sensing grating. Opt. Express.

[49] Noguchi Y (2019). Efficient measurement of the orbital-angular-momentum spectrum of an electron beam via a Dammann vortex grating. Phys. Rev. Appl..

[50] Gu XM (2018). Gouy phase radial mode sorter for light: concepts and experiments. Phys. Rev. Lett..

[51] Berkhout GCG (2010). Efficient sorting of orbital angular momentum states of light. Phys. Rev. Lett..

[52] Mirhosseini M (2013). Efficient separation of the orbital angular momentum eigenstates of light. Nat. Commun..

[53] Leach J (2002). Measuring the orbital angular momentum of a single photon. Phys. Rev. Lett..

[54] Leach J (2004). Interferometric methods to measure orbital and spin, or the total angular momentum of a single photon. Phys. Rev. Lett..

[55] Hickmann JM (2010). Unveiling a truncated optical lattice associated with a triangular aperture using light’s orbital angular momentum. Phys. Rev. Lett..

[56] Liu ZW (2019). Superhigh-resolution recognition of optical vortex modes assisted by a deep-learning method. Phys. Rev. Lett..

[57] Scholes S (2019). Structured light with digital micromirror devices: a guide to best practice. Optical Eng..

[58] Cox MA (2019). A high-speed, wavelength invariant, single-pixel wavefront sensor with a digital micromirror device. IEEE Access.

