# Supplementary material for: Divergence-degenerate spatial multiplexing towards future ultrahigh capacity, low error-rate optical communications
Source: Light Sci Appl. 2022 May 19;11:144. doi: 10.1038/s41377-022-00834-4 (PMC9117247; doi:10.1038/s41377-022-00834-4)
Supplement: Supplementary file 1 — Supplementary Information for Divergence-degenerate spatial multiplexing towards future ultrahigh capacity, low error-rate optical communications [file 41377_2022_834_MOESM1_ESM.pdf]

Supplementary Information for: Divergence-degenerate spatial multiplexing towards future ultrahigh capacity, low error-rate optical communications

Zhensong Wan[1,2], Yijie Shen[3], Zhaoyang Wang[1,2], Zijian Shi[1,2], Qiang Liu[1,2] and Xing Fu[1,2]

[1]*Key Laboratory of Photonic Control Technology (Tsinghua University), Ministry of Education, Beijing 100084, China*

[2]*State Key Laboratory of Precision Measurement Technology and Instruments, Department of Precision Instrument, Tsinghua University, Beijing 100084, China*

[3]*Optoelectronics Research Centre, University of Southampton, Southampton SO17 1BJ, UK*

Abstract: The supplementary information introduce the general multi-DoF ray-wave geometric beam (RWGB) as well as its orthogonality, beam quality, divergence and propagation in turbulence condition. In addition, the conjugate modulation identification method utilizing a digital micro-mirror device (DMD) is illustrated in detail. Furthermore, the 8-bit and 16-bit hybrid shift-keying encoding/decoding based on tri-DoF multi-vortex geometric beams (MVGBs) demultiplexing are demonstrated.

Supplementary Note 1: Multi-DoF ray-wave geometric beams and their orthogonality.

Ray-wave geometric beams. The Hermite-Gaussian (HG) eigenmodes are typical operating modes for a plano-concave laser resonator. The wave function of HG modes is given by [1, 2]:

$$\mathrm{HG}_{n,m,l} = \psi_n^{\mathrm{HG}}(\widetilde{x})\,\psi_m^{\mathrm{HG}}(\widetilde{y})\exp\left[ik_{n,m,l}\widetilde{z} - i(n+m+1)\,\theta_G\right] \quad \text{(S1)}$$

where $\theta_G = \tan^{-1}(z/z_R)$ is the Gouy phase, n and m are orders of transverse mode, l is the order of longitudinal mode, and the Hermite-Gaussian function is given by:

$$\psi_n^{\mathrm{HG}}(\widetilde{x}) = \frac{1}{\sqrt{2^n\sqrt{\pi}n!}}\frac{1}{\sqrt{\omega}}\exp\left(-\widetilde{x}^2/2\right)\mathrm{H}_n(\widetilde{x}) \quad \text{(S2)}$$

with

$$\widetilde{x} = \frac{\sqrt{2}x}{\omega}, \widetilde{y} = \frac{\sqrt{2}y}{\omega}, \widetilde{z} = z + \frac{\left(x^2+y^2\right)z}{2\left(z^2+z_R^2\right)}, k_{n,m,l} = \frac{\omega_{n,m,l}}{c} \quad \text{(S3)}$$

where the $\mathrm{H}_n(\cdot)$ represents the Hermite polynomials of n-th order, z_R is the Rayleigh length, $\omega = \omega_0\sqrt{1+(z/z_R)^2}$ is the beam waist and $\omega_0 = \sqrt{\lambda z_R/\pi}$ is the fundamental mode radius at the waist, λ is the wavelength of light and $k_{n,m,l}$ is the wave number.

In terms of the Wigner d-matrix, the eigenstates mode can be analytically expressed as a linear combination of the HG modes in Cartesian coordinate as:

$$\mathrm{HLG}_{n,m,l}^{(\alpha,\beta)} = \exp[\mathrm{i}(n+m)\alpha/2]\sum_{k=0}^{n+m}\mathrm{e}^{\mathrm{i}k\alpha}d_{k-\frac{n+m}{2},\frac{n-m}{2}}^{\frac{n+m}{2}}(\beta)\mathrm{HG}_{k,n+m-k,l} \quad \text{(S4)}$$

It should be noted, when $\beta = 0$ or $\beta = \pi$, $\mathrm{HLG}_{n,m,l}^{(\alpha,\beta)}$ are reduced into HG modes; and when $\alpha = \beta = \pm\pi/2$, LG modes are obtained. The elements of Wigner d-matrix are given by [3, 4]:

$$\begin{aligned} d_{k-\frac{n+m}{2},\frac{n-m}{2}}^{\frac{n+m}{2}}(\beta) &= \sqrt{k!(n+m-k)!n!m!n} \\ &\times \sum_{v=\max(0,k-n)}^{\min(m,k)} \frac{(-1)^v[\cos(\beta/2)]^{m+k-2v}[\sin(\beta/2)]^{n-k+2v}}{v!\,(m-v)!\,(k-v)!\,(n-k+v)!} \end{aligned} \quad \text{(S5)}$$

For a plano-concave cavity, the frequency-degenerate condition of coupled harmonic oscillators requires that $\Delta f_T/\Delta f_L = P/Q = 1/\pi\cos^{-1}\sqrt{1-L/R}$, where P and Q are integers, and $\Delta f_L(\Delta f_T)$ is the longitudinal (transverse) mode spacing. Under frequency-degenerate state with off-axis pumping, the SU(2) coherent state can be obtained [5, 6]. This ray-wave geometric beams (RWGBs) can be represented as the superposition of a family of eigenstates with sub-Poissonian distribution:

$$|\Psi_{n_0,m_0}^{(\alpha,\beta,\phi)}\rangle_{p,q}^{N} = \frac{1}{2^{N/2}}\sum_{K=0}^{N}\binom{N}{K}^{1/2}\mathrm{e}^{\mathrm{i}K\phi}\mathrm{HLG}_{n_0+pK,m_0+qK,l_0-PK}^{(\alpha,\beta)} \quad \text{(S6)}$$

where $N+1$ is the number of eigenmodes in frequency-degenerate family of $\mathrm{HLG}_{n_0+pK,m_0+qK,l_0-PK}^{(\alpha,\beta)}$, p and q are ratios of transverse frequency spaces in x- and y-axis, respectively, n_0 and m_0 are initial orders of transverse mode in x- and y-axis, respectively, l_0 is initial order of longitudinal mode in z-axis, and ϕ is the coherent-state phase. (α, β) are the two parametric rotation angles in SU(2) symmetry along z-axis. For simplicity, we use $\mathrm{HLG}_{n,m}^{(\alpha,\beta)}$ hereinafter and in the main text

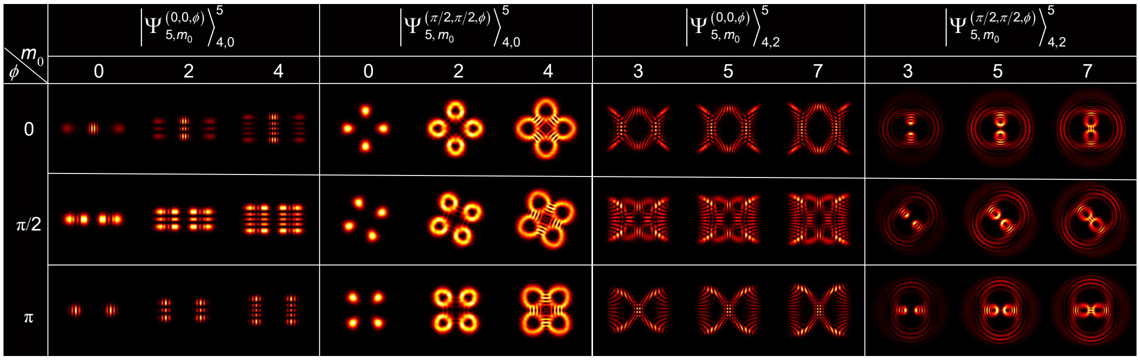

FIG. S1: Intensity patterns of ray-wave geometric beams. From left to right, the four areas are multi-path geometric beams, MVGBs, Lissajous and trochoidal parametric surface modes, respectively.

to represent the eigenstates from $\mathrm{HLG}^{(\alpha,\beta)}_{n,m,l}$, ignoring their longitudinal index$l$, which has no effect on the external modulation without optical cavity.

Orthogonality of general RWGBs. The intensity patterns of RWGBs, including multi-path geometric beams, multi-vortex geometric beams (MVGBs), Lissajous and Trochoidal parametric surfaces beams, are shown in order in Fig. S1. To utilize the multi-parameter space and realize effective mode identification, we study the correlation degree of two RWGBs on the degrees of freedom (DoFs) of $n_0(m_0)$, N and ϕ, as given by:

$$\begin{aligned}{}^{N_s}_{p,q}\left\langle \Psi^{(\alpha,\beta,\phi_s)}_{n_{0_s},m_{0_s}} \middle| \Psi^{(\alpha,\beta,\phi_r)}_{n_{0_r},m_{0_r}} \right\rangle^{N_r}_{p,q} = \iint \frac{1}{2^{N_s/2}} \sum_{J=0}^{N_s} \binom{N_s}{J}^{1/2} \mathrm{e}^{-\mathrm{i}J\phi_s} \widetilde{\mathrm{HLG}}^{(\alpha,\beta)}_{n_{0_s}+pJ,m_{0_s}+qJ} \\ \times \frac{1}{2^{N_r/2}} \sum_{K=0}^{N_r} \binom{N_r}{K}^{1/2} \mathrm{e}^{\mathrm{i}K\phi_r} \mathrm{HLG}^{(\alpha,\beta)}_{n_{0_r}+pK,m_{0_r}+qK} dxdy\end{aligned} \tag{S7}$$

where sign '∼' means conjugate, and $\iint \widetilde{\mathrm{HLG}}^{(\alpha,\beta)}_{n_s,m_s} \times \mathrm{HLG}^{(\alpha,\beta)}_{n_r,m_r} dxdy = \delta_{(n_s,n_r),(m_s,m_r)}$, where $\delta_{(n_s,n_r),(m_s,m_r)} \neq 0$ only when $n_s = n_r$ and $m_s = m_r$. Two RWGBs are orthogonal to each other when the correlation degree is zero.

First, we consider the case of two RWGBs with different parameters of n_0 and m_0. When $n_{0_s} - n_{0_r} \neq Zp$ or $m_{0_s} - m_{0_r} \neq Zq$ (where Z is an arbitrary integer), the family of eigenmodes of $|\Psi^{(\alpha,\beta,\phi)}_{n_{0_s},m_{0_s}}\rangle^N_{p,q}$ and $|\Psi^{(\alpha,\beta,\phi)}_{n_{0_r},m_{0_r}}\rangle^N_{p,q}$ are totally different and orthogonal to each other. The theoretical correlation degree between $|\Psi^{(\pi/2,\pi/2,0)}_{10,0}\rangle^5_{5,0}$ and $|\Psi^{(\pi/2,\pi/2,0)}_{n_0,0}\rangle^5_{5,0}$ where n_0 is changing from 0 to 30 are shown in the first row of Fig. S2 (a).

For the case of two RWGBs with only different parameter of N, Eq. S7 is reduced to:

$${}^{N_s}_{p,q}\left\langle \Psi^{(\alpha,\beta,\phi)}_{n_0,m_0} \middle| \Psi^{(\alpha,\beta,\phi)}_{n_0,m_0} \right\rangle^{N_r}_{p,q} = \frac{1}{2^{\frac{N_s+N_r}{2}}} \sum_{K=0}^{\min(N_s,N_r)} \binom{N_s}{K}^{1/2} \binom{N_r}{K}^{1/2} \tag{S8}$$

where the correlation degree of two RWGBs with different N is not zero, meaning non-orthogonal. The theoretical correlation degree between $|\Psi^{(\pi/2,\pi/2,0)}_{5,5}\rangle^{10}_{3,0}$ and $|\Psi^{(\pi/2,\pi/2,0)}_{5,5}\rangle^{N}_{3,0}$ where N is changing from 0 to 25 are shown in the first row of Fig. S2 (b). One can see the correlation degree is

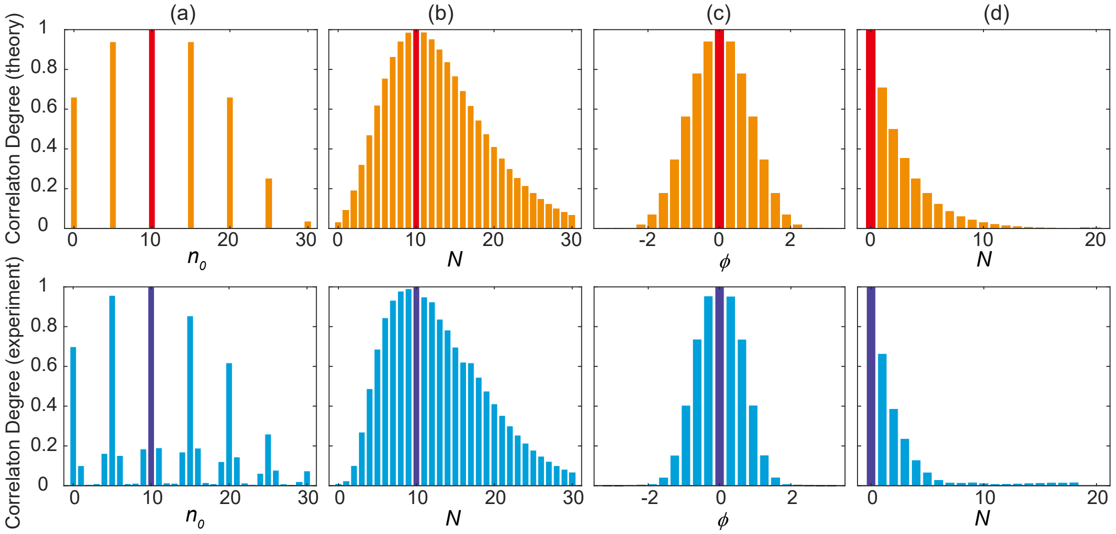

FIG. S2: Correlation degree of RWGBs in theory and experiments.
(a) Correlation degree between $|\Psi_{10,0}^{(\pi/2,\pi/2,0)}\rangle_{5,0}^{5}$ and $|\Psi_{n_0,0}^{(\pi/2,\pi/2,0)}\rangle_{5,0}^{5}$ where n_0 is changing from 0 to 30,
(b) Correlation degree between $|\Psi_{5,10}^{(\pi/2,\pi/2,0)}\rangle_{5,0}^{10}$ and $|\Psi_{5,10}^{(\pi/2,\pi/2,0)}\rangle_{5,0}^{N}$ where N is changing from 0 to 25,
(c) Correlation degree between $|\Psi_{5,10}^{(\pi/2,\pi/2,0)}\rangle_{5,0}^{5}$ and$|\Psi_{5,10}^{(\pi/2,\pi/2,\phi)}\rangle_{5,0}^{5}$ where ϕ is changing from $-\pi$ to π,
(d) Correlation degree between $|\Psi_{5,10}^{(\pi/2,\pi/2,0)}\rangle_{5,0}^{5}$ and $|\Psi_{5,10}^{(\pi/2,\pi/2,\pi/2)}\rangle_{5,0}^{N}$ where N is changing from 0 to 20.
The dark bar in each subfigure indicates the reference index value for the correlation degree analysis.

close to zero when the N indices of two modes are far enough apart, the condition of which can be considered as quasi-orthogonal. However, the parameter N influences the correlation degree of parameter ϕ, thus it is not introduced as an independent DoF in the main text.

For the case of two RWGBs with only different parameter of ϕ, Eq. S7 is reduced to:

$$ {}_{p,q}^{N}\left\langle \Psi_{n_0,m_0}^{(\alpha,\beta,\phi_r)} \middle| \Psi_{n_0,m_0}^{(\alpha,\beta,\phi_s)} \right\rangle_{p,q}^{N} = \frac{1}{2^N} \sum_{K=0}^{N} \binom{N}{K} \exp\left[\mathrm{i}K\left(\phi_r - \phi_s\right)\right] \tag{S9} $$

where the correlation degree depends on the difference of two coherent-state phases $(\phi_r - \phi_s)$ as well as N. Here, we examine the cases where the phase difference are π and $\pi/2$. The theoretical correlation degree between $|\Psi_{5,10}^{(\pi/2,\pi/2,0)}\rangle_{5,0}^{5}$ and$|\Psi_{5,10}^{(\pi/2,\pi/2,\phi)}\rangle_{5,0}^{5}$ where ϕ is changing from $-\pi$ to π are shown in the first row of Fig. S2 (c). First, when $\phi_r - \phi_s = \pi$, Eq. S9 can be reduced to $\frac{1}{2^N}\sum_{K=0}^{N}(-1)^K\binom{N}{K} = 0$, meaning that two RWGBs are orthogonal to each other. However, when $\phi_r - \phi_s = \pm\pi/2$, Eq. S9 can be reduced to $\frac{1}{2^N}\sum_{K=0}^{N}(\pm i)^K\binom{N}{K}$, in which the correlation degree of two RWGBs depends on N. The theoretical correlation degree between $|\Psi_{5,10}^{(\pi/2,\pi/2,0)}\rangle_{3,2}^{5}$ and

$|\Psi_{5,10}^{(\pi/2,\pi/2,\pi/2)}\rangle_{3,2}^{N}$ where N is changing from 0 to 20 are shown in the first row of Fig. S2 (d). The correlation degree decreases rapidly as N increases when the difference of coherent-state phase is $\pi/2$, in which the case with a small correlation degree (smaller than 0.1 when $N \geqslant 5$) can be considered as quasi-orthogonal. The experimental results of correlation degree related to spatial indices of n_0, N and ϕ are shown in the second row of Fig. S2. One can see all the experimental results agree well with the theoretical results, with the root mean square errors of 0.0049, 0.0053, 0.016 and 0.0012, respectively.

Divergence and propagation of MVGBs. The beam quality factor M^2 is an important metric of the divergence of a superposed high-order transverse mode, and an indicator that characterizes whether the transmission behavior is consistent[7]. Modes with the same M^2 would propagate in an identical manner as they have the same space-bandwidth product. MVGBs as a superposition of multiple high-order eigenmodes have their propagating behavior in free space completely determined by the component weights. As noted in the main text, the beam quality factor of the MVGB depends entirely on the family of eigenmodes it contains and the corresponding normalized weighting factor. As Fig. S3 shows, there are nine M^2 factors being shared by 100 orthogonal MVGBs. Meanwhile, there are twenty-eight M^2 factors being shared by 100 LG beams. For instance, among the 100 lowest orders of MVGB modes as a set of densely packed information carriers, by combinations of m_0=$\{0, 1, 2, 3, 4\}$, n_0=$\{0, 1, 2, 3, 4\}$, and ϕ=$\{0, \pi/2, \pi, 3\pi/2\}$, the maximum beam quality degeneracy reaches as high as 20 that up to 20 modes share the same beam quality factor of M^2=17.5. This leads to only a total of 9 beam quality factors from all the 100 modes: M^2=$\{13.5, 14.5, 15.5, 16.5, 17.5, 18.5, 19.5, 20.5, 21.5\}$. In contrast, the 100 lowest orders of LG_p^l modes, by combinations of p and l both taking 10 integer values from 0 to 9, have 28 integer values of M^2 from 1 to 28. Meanwhile, the second moment of intensity of any order LG beams or MVGBs can be used for the calculations of beam cross-section diameter D. Waist diameters of LG modes and MVGBs in the 100 lowest orders, normalized to those of fundamental modes, are shown in Fig. S4. Furthermore, Fig. S5 illustrates the simulated spot sizes at the receiver over a 2 km link as a function of transmitted beam size, for OAM beams and MVGBs with various different M^2,

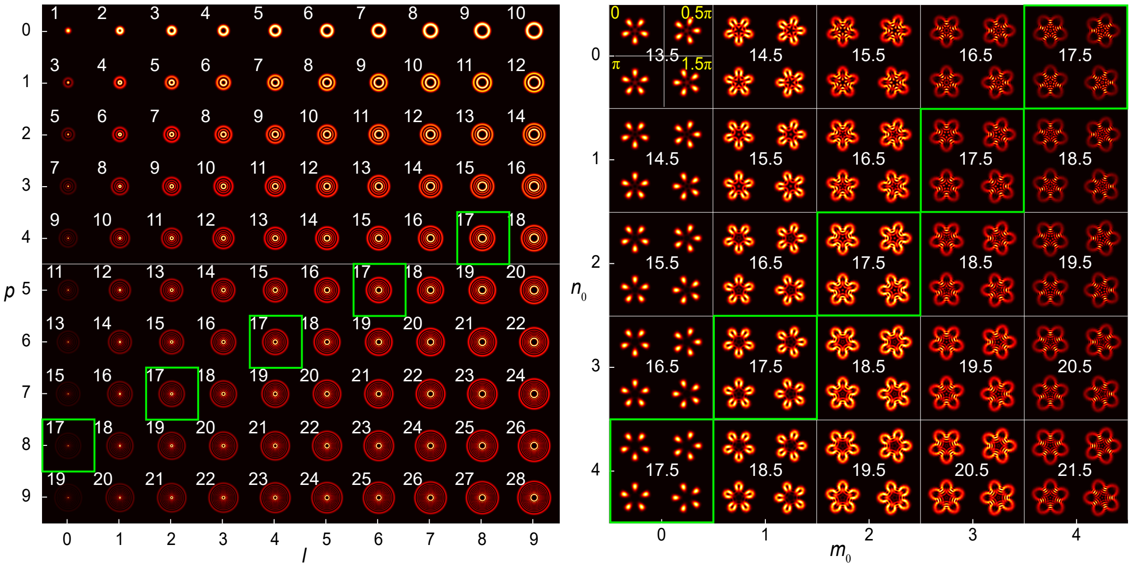

FIG. S3: Intensity patterns and beam quality factor (M^2) of LG modes in 100 lowest orders (left) and MVGBs in 100 lowest orders (right).

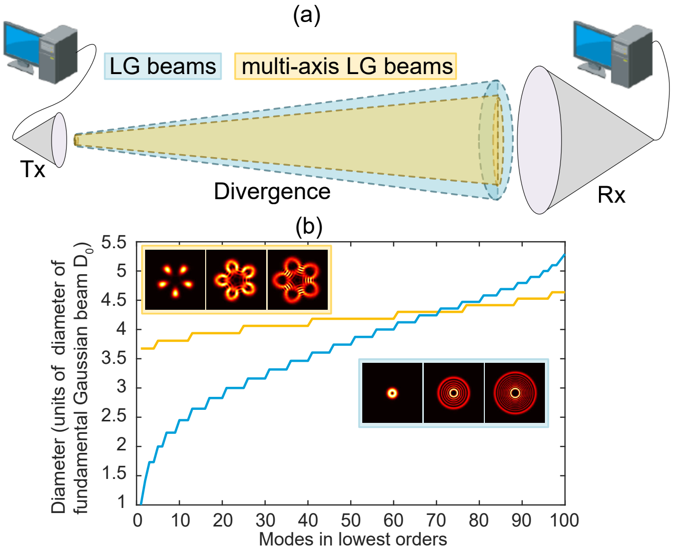

FIG. S4: Divergence contrast between LG modes from LG_0^1 to $\mathrm{LG}_9^1$0(blue) and MVGBs from $|\Psi_{3,0}^{(\pi/2,\pi/2,0)}\rangle_{5,0}^{5}$ to $|\Psi_{7,4}^{(\pi/2,\pi/2,3\pi/2)}\rangle_{5,0}^{5}$ (orange): (a) schematic of beam divergence in free space communication system; (b) normalized beam waist diameters of MVGBs and LG modes in 100 lowest orders

in which the corresponding minimum spot sizes at the receiver for all cases are marked as stars.

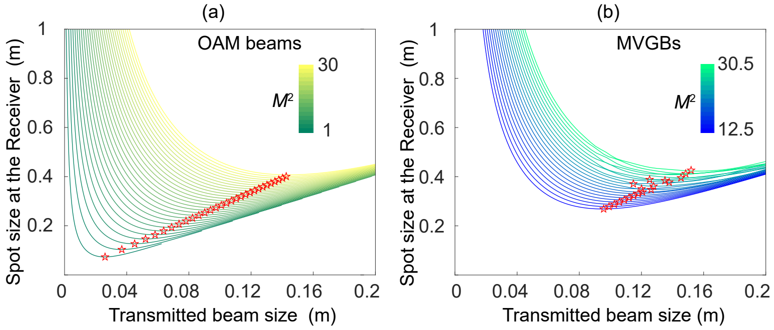

FIG. S5: Simulated spot sizes at the receiver over a 2 km link as a function of transmitted beam size, for OAM beams and MVGBs

To investigate the free-space propagating performance of MVGBs against perturbation caused by atmospheric turbulence, we run a simulation that uses the Modified von Karman turbulence model[8], assuming the strength of optical turbulence as C_n^2=5×10^{-15} that corresponds to a moderate turbulence level, the atmospheric coherence diameter of r_0=0.5 m, the propagation distance mesh of Δz=40 m, the simulation area size of 0.6 m×0.6 m, and the outer and inner scale of turbulence as 500 m and 0.01 m, respectively. Fig. S6 demonstrates the simulated results averaging 10 trials of the power portion that remains in the original channel over a free-space propagation link up to 2 km, in the presence of a moderate level of atmospheric turbulence, for both cases of MVGBs and LG beams. Note that subfigures (a) and (b) only show the channel proportions of five main components respectively, while all the components are described in the histograms in subfigures (a1-a4, b1-b4). It can be seen that the MVGBs and LG beams have approximate signal

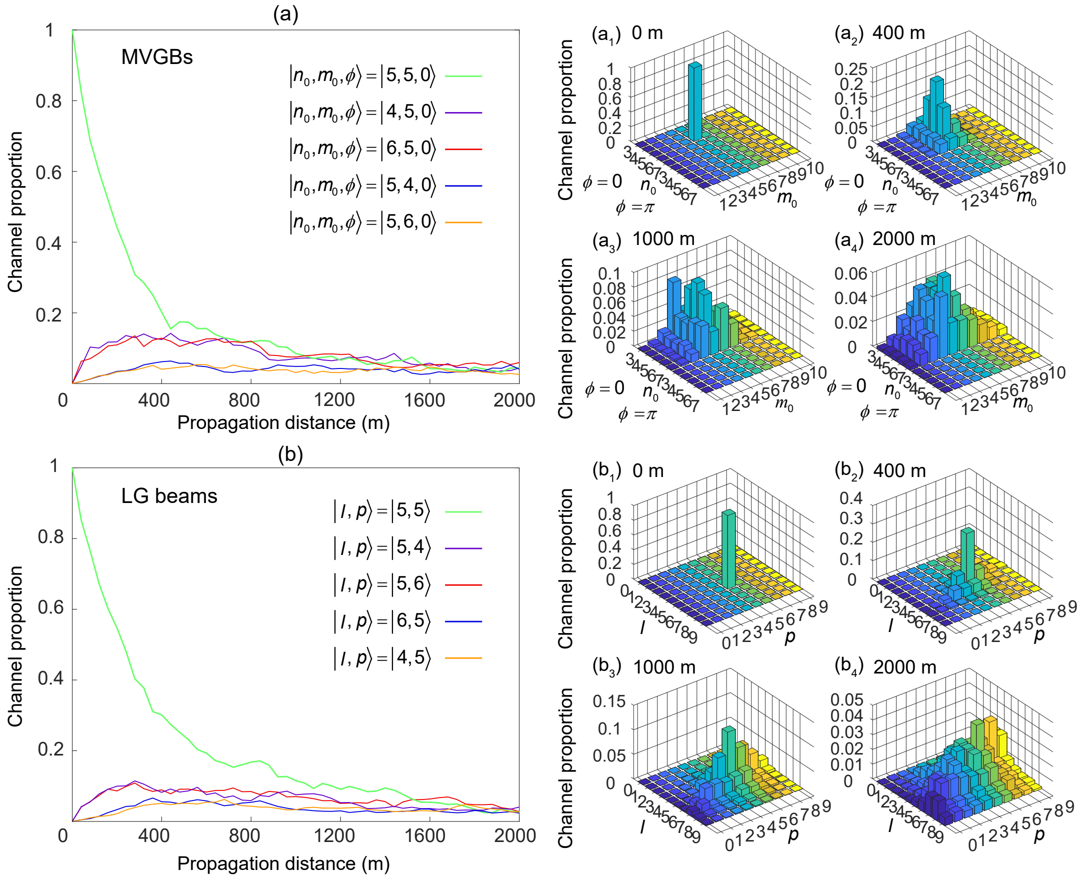

FIG. S6: Free-space propagating performance against atmospheric turbulence for the cases of MVGB of $|\Psi_{5,5}^{(\pi/2,\pi/2,0)}\rangle_{5,0}^{5}$ and LG_5^5.

power loss due to modal power coupling effect, which can be effectively mitigated by adaptive optics. Note that the DoF of coherent-state phase of the MVGBs is not disturbed by turbulence at all, which may be exploited in the practical applications.

Supplementary Note 2: Conjugated modulation demultiplexing method for RWGBs.

The design of conjugated holographic mask (CHM) of RWGBs is presented by utilizing the complex amplitude modulation approach [9, 10]. The transverse mode of a MVGB can be simplified to a normalized complex scalar field:

$$SU(x, y) = A(x, y) \exp[\mathrm{i}\Phi(x, y)]\,. \tag{S10}$$

where $A(x, y) \in [0, 1]$ is normalized amplitude distribution, $\Phi(x, y) \in [0, 2\pi]$ is phase distribution. The conjugated optical field of RWGB is $\widetilde{SU}(x, y) = A(x, y) \exp[-\mathrm{i}\Phi(x, y)]$. The DMD

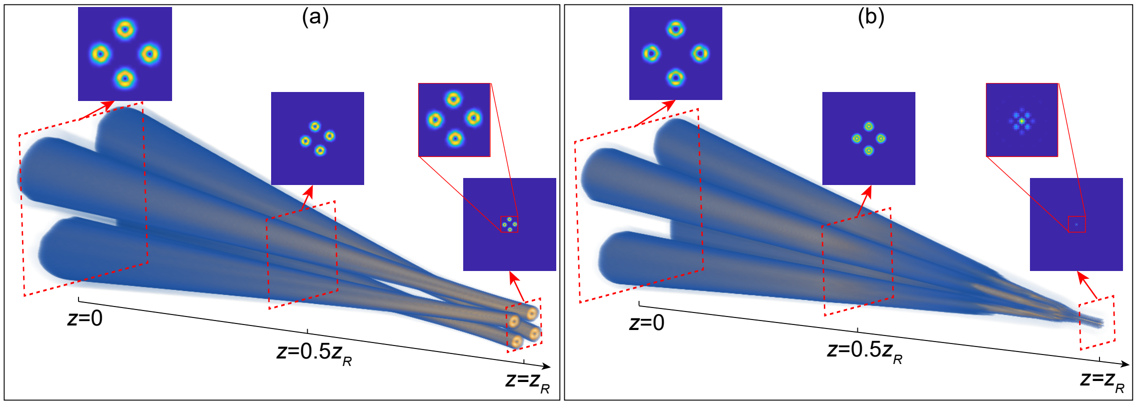

FIG. S7: Theoretical propagating evolution of wave packet from the focusing lens with the focal length of f to the Fourier plane: (a) normal MVGB; (b) conjugated modulated MVGB

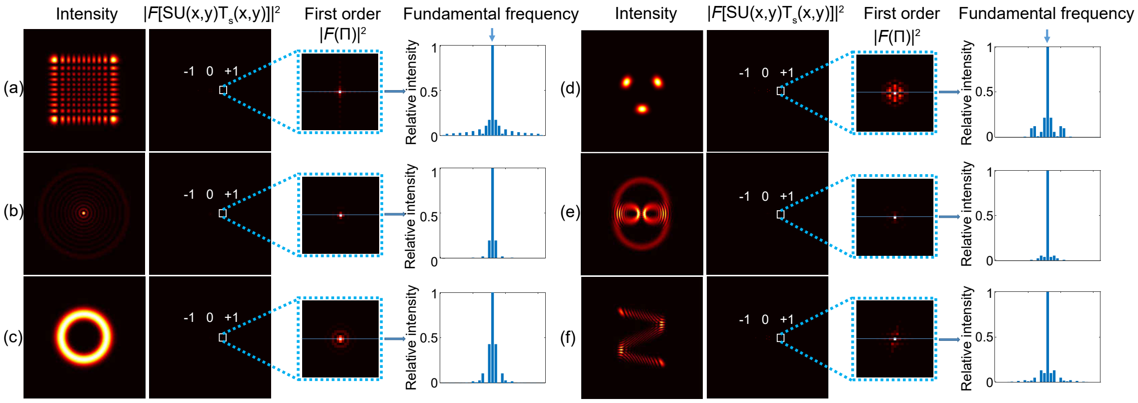

FIG. S8: Examples of mode identification by conjugated modulation method. (a) $\mathrm{HG}_{10,10}$; (b) $\mathrm{LG}_{p=10}^{l=0}$; (c)$\mathrm{LG}_{p=0}^{l=10}$; (d) $|\Psi_{10,0}^{(0,0,0)}\rangle_{3,0}^{5}$; (e) $|\Psi_{10,10}^{(\pi/2,\pi/2,0)}\rangle_{1,3}^{5}$; (f) $|\Psi_{10,10}^{(0,0,0)}\rangle_{1,3}^{5}$. In each subfigure, the first column shows intensity patterns of eigenmodes and general RWGBs, the second column shows the Fourier transformation of intensity, and the third and last columns are enlarged views of the first order spectrum.

transmission function of the hologram for this conjugated optical field is given as:

$$\begin{aligned} T\left(x,y\right) &= Mask(A,-\Phi) \\ &= \frac{1}{2}+\frac{1}{2}\mathrm{sign}\left\{\cos\left[-\Phi(x,y)\right]+\cos\left[\arcsin A(x,y)\right]\right\} \end{aligned} \tag{S11}$$

Now we have obtained the target conjugated mode in the first order, which needs to be separated from the other orders. A simple way of separating the mode is to add different spatial frequencies, e.g. a linear grating, into different Fourier series. The conjugated optical fields are given by $\widetilde{SU}_s\left(x,y\right)=A\left(x,y\right)\exp\left[-\mathrm{i}\Phi\left(x,y\right)+2\pi\left(u_0x+v_0y\right)\right]$, and the CHM is given by:

$$T_s\left(x,y\right)=Mask[A,-\Phi+2\pi\left(u_0x+v_0y\right)] \tag{S12}$$

where the u_0 and v_0 are the reciprocal of the period of linear grating in x and y direction, respectively. The identified RWGBs illuminate the corresponding CHMs, which can be represented as

$\Omega = SU(x,y) \times T_s(x,y)$. In the first order of Fourier series, we have:

$$\Pi = SU\,(x,y)\,\boldsymbol{.}\,\widetilde{SU}\,(x,y) = A(x,y)^2 \tag{S13}$$

where Π is the product of the identified RWGBs and its conjugated field, corresponding to the intensity distribution of the identified light field. The far-fields of the regular OAM beams and RWGBs have a hollow structure and a complex spatial distribution respectively, which means the spatial spectrum of the zero frequency is small or even non-existent. However, Π is a real function. Therefore, the Fourier transform of the first order diffraction can obtain the fundamental frequency dominance result that the direct Fourier transform of the light field cannot obtain, which is vividly illustrated in Fig. S7, comparing the theoretical propagating evolution of wave packet of a normal RWGB and a conjugated RWGB, from the focusing lens to the Fourier plane. The important feature of fundamental frequency dominance enables the conjugated modulation method to effectively identify RWGBs. The Fourier transform or far-field of first order Fourier series is given as:

$$F\,(\Pi) = F\left[A(x,y)^2\right] \tag{S14}$$

CHMs are designed for identifying eigenmodes (HG and LG modes) and RWGBs (MVGBs, Lissajous, and Trochodial parametric beams). Fig. S8 (a-c) demonstrate the conjugated modulation simulated results of $\mathrm{HG}_{10,10}$, $\mathrm{LG}_{p=10}^{l=0}$ and $\mathrm{LG}_{p=0}^{l=10}$, in which the third and fourth rows show that the spatial fundamental frequency of first order is obviously stronger than the other frequencies. However, there are almost no energy in the central spectral region of original complex optical field, which implies the fundamental frequency can be easily discerned. Similarly, the simulation results of conjugated modulation of the $|\Psi_{10,0}^{(0,0,0)}\rangle_{3,0}^{5}$, $|\Psi_{10,10}^{(\pi/2,\pi/2,0)}\rangle_{1,3}^{5}$, and $|\Psi_{10,10}^{(0,0,0)}\rangle_{1,3}^{5}$ are shown in Fig. S8 (d-f).

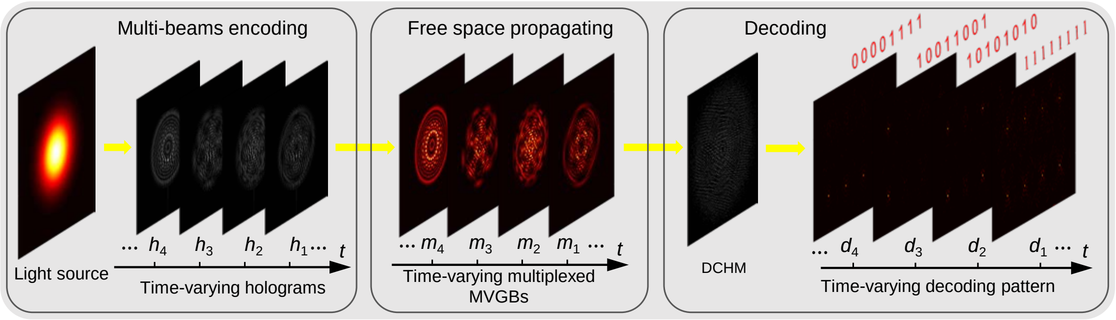

FIG. S9: Schematic of data transmission based on multi-MVGB shift-keying, Demultiplexed CHM: demultiplexed conjugated holographic mask.

Supplementary Note 3: High-dimensional hybrid shift-keying encoding/decoding.

Based on the demultiplexing approach of MVGBs in the main text, we introduce and demonstrate the concept of high-dimensional hybrid shift-keying encoding, using tri-DoF MVGBs as an information carrier for time-varying signal encoding to extend the signal transmission channel. A flow chart of 8-bit MVGBs shift-keying encoding/decoding is shown in Fig. S9 corresponding to the experimental setup in Fig. 7 in main text. First, a time-varying multiplexed MVGBs sequence

is obtained, by the holograms loaded on DMD #1 according to the encoded signal. After free-space propagation, the coded multiplexed MVGBs illuminate DMD #2 loaded with a constant demultiplexed CHM, and are separated into 8 diffraction positions, with the focal spots recorded by CCD. Finally, the signal can be decoded by the sorting of MVGBs.

The experimental results of 8-bit signal decoding are shown in Fig. S10. Eight MVGBs that serve as information carriers are $|\Psi^{(\pi/2,\pi/2,\phi)}_{5,m_0}\rangle^{5}_{5,0}$, where the values of two employed DoFs (ϕ, m_0) are $(0, 8)$, $(\pi/2, 8)$, $(\pi, 8)$, $(3\pi/2, 8)$, $(0, 10)$, $(\pi/2, 10)$, $(\pi, 10)$ and $(3\pi/2, 10)$. The corresponding demultiplexed CHM is $\sum_{\phi,m_0} |\widetilde{\Psi}^{(\pi/2,\pi/2,\phi)}_{5,m_0}\rangle^5_{5,0} \exp\left[\mathrm{i}2\pi(u_\phi x + v_{m_0} y)\right]$. The parameters ϕ and m_0 are distinguished in x and y directions, respectively. Fig. S10 (a)-(h) show the 8-bit decoding patterns of eight numbers (1, 21, 91, 125, 135, 185, 215 and 255), respectively. It can be seen the signal is well recovered with zero BER. In a further step to demonstrate 16-bit case, $|\Psi^{(\pi/2,\pi/2,\phi)}_{7,m_0}\rangle^{5}_{5,0}$ is added as the signal carrier, while the set of (ϕ, m_0) is same as that of 8-bit case. The corresponding demultiplexed CHM is $\sum_{\phi,m_0,n_0} |\widetilde{\Psi}^{(\pi/2,\pi/2,\phi)}_{n_0,m_0}\rangle^5_{5,0} \exp\left\{\mathrm{i}2\pi[u_\phi x + (v_{m_0} + w_{n_0})y]\right\}$. The experimental results of 16-bit signal decoding for 10001, 20001, 40001 and 50001 as shown in Fig. S11 (a)-(d) demonstrate an excellent performance as well. The maximum refresh rate of DMD used is about 11 kHz, indicating the highest transmission rates for 8-bit and 16-bit cases are $8\times11\mathrm{k} = 0.88$ Mbit/s and $16\times11\mathrm{k} = 1.76$ Mbit/s, respectively. The data is recorded at 10 Hz, as restricted by the collection rate of CCD.

We have implemented the tri-DoF MVGBs as information carrier for the first time, demonstrating 8/16-bit MVGBs hybrid shift-keying without error, as well as multi-channel multiplexing communication with low cross-talk. Meanwhile, the DMD modulation technology make the information encoding speed two to three orders of magnitude faster than the SLM encoding of OAM beams, which make the MVGBs encoding feasible in the communication system. In addition to shift-keying approach as demonstrated, MVGBs can also be used for multi-channel mode multiplexing spatial optical communications, as verified by results of Fig. S10 and Fig. S11, which is fully compatible with wavelength and polarization division multiplexing.

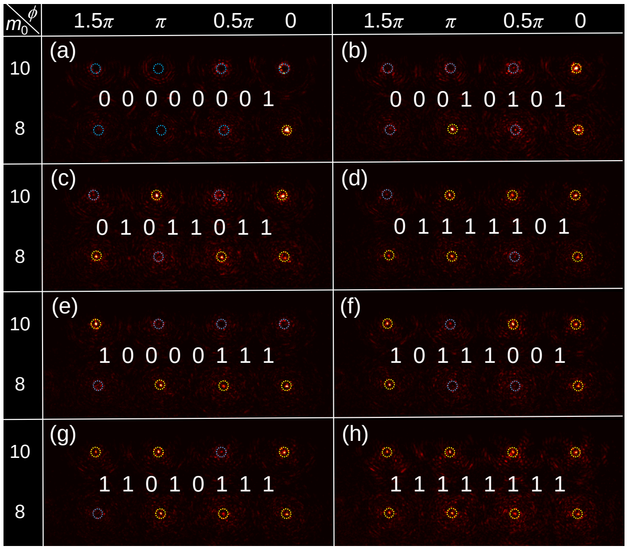

FIG. S10: The decoding results of 8-bit MVGB shift-keying, (a-h) are 8-bit binary code of [1, 21, 91, 125, 135, 185, 215, 255].

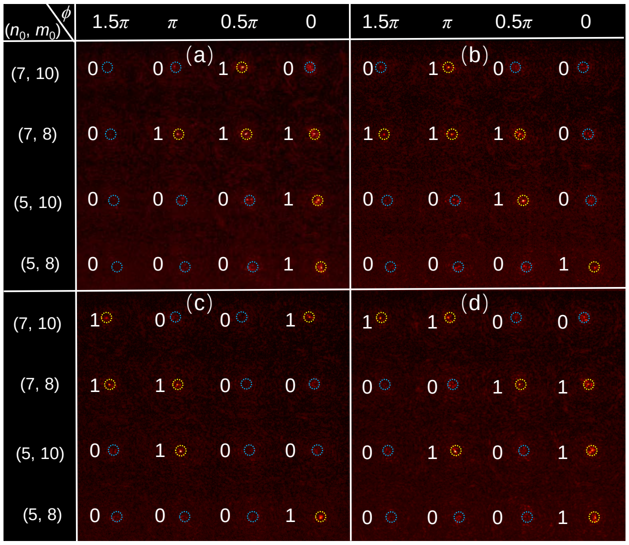

FIG. S11: The decoding results of 16-bit MVGBs shift-keying, (a-f) are 16-bit binary code of [10001, 20001, 40001, 50001].

[1] Abramochkin, E. G. & Volostnikov, V. G. Generalized Hermite-Laguerre-Gauss beams. *Physics of Wave Phenomena* **18**, 14–22 (2010).

[2] Wan, Z., Shen, Y., Gong, M. & Fu, X. Quadrant-separable multi-singularity vortices manipulation by coherent superposed mode with spatial-energy mismatch. *Optics Express* **26**, 34940 (2018).

[3] Chen, Y. F., Tung, J., Tuan, P. & Huang, K.-F. Symmetry breaking induced geometric surfaces with topological curves in quantum and classical dynamics of the SU(2) coupled oscillators. *Annalen der Physik* **529**, 1600253 (2017).

[4] Chen, Y. F. Geometry of classical periodic orbits and quantum coherent states in coupled oscillators with SU(2) transformations. *Physical Review A* **83**, 032124 (2011).

[5] Blumel, R. *Advanced quantum mechanics: the classical-quantum connection* (Jones & Bartlett Publishers, 2011).

[6] Hartley, J. G. & Ray, J. R. Coherent states for the time-dependent harmonic oscillator. *Physical Review D* **25**, 382 (1982).

[7] Phillips, R. L. & Andrews, L. C. Spot size and divergence for laguerre gaussian beams of any order. *Applied Optics* **22**, 643–644 (1983).

[8] Schmidt, J. D. Numerical simulation of optical wave propagation: With examples in matlab (SPIE, 2010).

[9] Scholes, S., Kara, R., Pinnell, J., Rodríguez-Fajardo, V. & Forbes, A. Structured light with digital micromirror devices: a guide to best practice. *Optical Engineering* **59**, 1 (2019).

[10] Cox, M. A., Toninelli, E., Cheng, L., Padgett, M. J. & Forbes, A. A high-speed, wavelength invariant, single-pixel wavefront sensor with a digital micromirror device. *IEEE Access* **7**, 85860–85866 (2019).
